# Curcumin Protects Neurons from Glutamate-Induced Excitotoxicity by Membrane Anchored AKAP79-PKA Interaction Network

**DOI:** 10.1155/2015/706207

**Published:** 2015-06-10

**Authors:** Kui Chen, Yu An, Lu Tie, Yan Pan, Xuejun Li

**Affiliations:** ^1^State Key Laboratory of Natural and Biomimetic Drugs, Department of Pharmacology, School of Basic Medical Sciences, Peking University, Beijing 100191, China; ^2^Beijing Key Laboratory of Tumor Systems Biology, Peking University, Beijing 100191, China

## Abstract

Now stimulation of AMPA receptor as well as its downstream pathways is considered as potential central mediators in antidepressant mechanisms. As a signal integrator which binds to AMPA receptor, A-kinase anchoring protein 79-(AKAP79-) PKA complex is regarded as a potential drug target to exert neuroprotective effects. A well-tolerated and multitarget drug curcumin has been confirmed to exert antidepressant-like effects. To explore whether AKAP79-PKA complex is involved in curcumin-mediated antiexcitotoxicity, we detected calcium signaling, subcellular location of AKAP79-PKA complex, phosphorylation of glutamate receptor, and ERK and AKT cascades. In this study, we found that curcumin protected neurons from glutamate insult by reducing Ca^2+^ influx and blocking the translocation of AKAP79 from cytomembrane to cytoplasm. In parallel, curcumin enhanced the phosphorylation of AMPA receptor and its downstream pathways in PKA-dependent manner. If we pretreated cells with PKA anchoring inhibitor Ht31 to disassociate PKA from AKAP79, no neuroprotective effects were observed. In conclusion, our results show that AKAP79-anchored PKA facilitated the signal relay from AMPA receptor to AKT and ERK cascades, which may be crucial for curcumin-mediated antiexcitotoxicity.

## 1. Introduction

To clarify the pathogenesis of major depressive disorder (MDD) and increase effective rate of antidepressant treatment is the direction of all the psychiatrists in the world. Today, the situation of antidepressant treatment is not optimistic, because the response rate of current antidepressants is just 60–70% and the clinical remission rate is only about 30% which suggests the imbalance of monoamine neurotransmitters may not be the key pathogenesis for MDD [1]. Currently, the orientation of antidepressant development is mainly focused on NMDA receptor antagonists due to its rapid and long-lasting antidepressant effects [2]. Blocking NMDA receptors can not only inhibit excessive glutamate-mediated activation of extrasynaptic NMDA receptor but also enhance AMPA receptor signal transduction to exert antidepressant effects [3]. In view of the popular abnormal glutamate receptors hypothesis, glutamate-induced excitotoxicity is increasingly used as cell model of MDD, which is characterized by glutamate receptor excessive activation and calcium overload [4–6].

Interestingly, cAMP-PKA cascade has been reported to associate with pathophysiology of MDD and ketamine-mediated antidepressant actions [7]. Reduced PKA activity has been observed in depressed patients and antidepressants can upregulate PKA activity [8]. In vitro studies also found that PKA activators showed the antidepressant-like effects in animal model of depression, while PKA activators mediated antidepressant effects could be totally blocked by PKA inhibitor, suggesting PKA may serve as a new drug target for depression treatment [9, 10]. The A-kinase anchoring proteins (AKAPs) are signal-assembling hub which can target various enzymes in the appropriate compartment. Notably, AKAPs have high affinity to the regulatory subunit of PKA and anchor PKA in the precise subcellular location. In the brain, AKAP79 is the main AKAP subunit which can direct PKA toward AMPA receptor subunit GluR1 in neuronal postsynaptic membrane. So theoretically, it is reasonable to speculate that AKAP79-PKA complex may be involved in the antidepressant mechanisms of NMDAR antagonists or PKA enhancers.

As a polyphenolic natural product, curcumin has already been confirmed to have antiexcitotoxicity effects [11]. In addition, curcumin has also been demonstrated to show antidepressant-like effects in MDD animal models, which are exposed to chronic unpredictable mild stress (CUMS) [12]. Considering that curcumin has a range of drug targets and can influence numerous signal transmissions, to find the specific and key mechanism of curcumin-mediated antidepressant effects is extremely urgent. In this paper, we employed SH-SY5Y human neuroblastoma cells as the experimental model for glutamate excitotoxicity, and all experiments reported here were designed to evaluate whether AKAP79-PKA complex participated in curcumin-mediated neuroprotective effects as the crucial molecular mechanism.

## 2. Materials and Methods

### 2.1. Reagents

Curcumin and glutamate were purchased from Sigma-Aldrich (St. Louis, MO, USA). The LDH assay kit was from Nanjing Jiancheng Bioengineering Institute (Nanjing, China). Antibodies to MEK1/2, p-MEK1/2, ERK1/2, p-ERK1/2, AKT, and p-AKT were obtained from Cell Signaling Technology (Beverly, MA, USA). Antibodies to PKA, NR1, p-NR1, GluR1, p-GluR1, GAPDH, Na^+^-K^+^-ATPase *α*1, and rabbit IgG were purchased from Bioworld (Louis Park, MN, USA). AKAP79 antibody and Protein A/G-Agarose were from Santa Cruz Biotechnology (Santa Cruz, CA, USA). Alkaline phosphatase-conjugated secondary antibodies were purchased from PIERCE. BCIP (3-bromo-4-chloro-5-indolyl phosphate) and NBT (nitro blue tetrazolium) were obtained from Ameresco. DyLight 488 and DyLight 594 conjugated secondary antibodies were bought from Jackson ImmunoResearch Laboratories (West Grove, PA, USA). The MTS assay kit was bought from Promega. The calcium indicator dye fluo-3 was bought from Beyotime Biotechnology. The BCA assay kit and chamber coverslip were purchased from Thermo Fisher Scientific (Rockford, IL). Fetal bovine serum was from HyClone (Logan, UT). Protease inhibitor cocktails were purchased from Roche (Basel, Switzerland).

### 2.2. Drug Treatment

Curcumin was dissolved in dimethyl sulfoxide (DMSO, ≤0.01%) [13, 14]. The PKA anchoring inhibitor Ht31 and control fragment Ht31p (both 20 *μ*M) were pretreated for 45 min before the administration of curcumin [15, 16]. Glutamate was dissolved in Locke's buffer (154 mM NaCl, 5.6 mM KCl, 3.4 mM NaHCO_3_, 1.2 mM MgCl_2_, 5.6 mM glucose, 5 mM HEPES, and 2.3 mM CaCl_2_, pH 7.4) [4]. Neurons were exposed to 30 mM glutamate for 12 hours to induce excitotoxicity.

### 2.3. Measurement of Intracellular Free Ca^2+^ Concentration

Intracellular free Ca^2+^ levels ([Ca^2+^]_*i*_) were quantified by fluorescence imaging of the calcium indicator dye fluo-3. Cells were incubated for 30 min in the presence of 5 *μ*M fluo-3 and then washed twice to remove extracellular dye. Cells were further incubated for 30 min prior to imaging. The fluorescence intensity was monitored by Zeiss microscope. The [Ca^2+^]_*i*_ in 10–15 neuronal cell bodies per microscope field was monitored prior to and after exposure of cells to glutamate insult.

### 2.4. Cell Culture

SH-SY 5Y cells were purchased from the Cell Culture Centre of Institute of Basic Medical Science, Chinese Academy of Medical Sciences. Briefly, the cells were cultured in DMEM medium (high glucose, no glutamine) supplemented with 2 mM L-glutamine, fetal bovine serum 10% (growth medium for cell proliferation) or 2.5% (maintenance medium for drug treatment, glutamate insult, and follow-up experiments), and antibiotics (penicillin and streptomycin) at 37°C in a humidified atmosphere consisting of 95% air/5% CO_2_. Upon reaching 75% confluence, the cells were exposed to either drugs or vehicle and then collected and extracted by RIPA lysis buffer (50 mM Tris-HCl (pH 7.4), 150 mM NaCl, 1% NP-40, and 0.1% SDS) containing 1% PMSF and protease inhibitor cocktails. The lysates were centrifuged at 12,000 rpm for 20 minutes at 4°C. The supernatants were collected and stored at −80°C. Sample protein concentrations were determined by BCA assay.

### 2.5. MTS Assay

SH-SY 5Y cells were plated in 96-well plates for 24 hours. In accordance with our previous research, pretreatment of 1 *μ*M curcumin for 6 hours was employed in the present study and 30 mM glutamate was incubated with neurons for 12 hours [4, 17, 18]. After incubation with drugs, cell viabilities were determined by MTS (3-(4,5-dimethylthiazol-2-yl)-5-(3-carboxymethoxyphenyl)-2-(4-sulfophenyl)-2H-tetrazolium, inner salt) assay. After three washes with PBS, the cells were exposed to 100 *μ*L new media containing 20 *μ*L MTS at 37°C for 3 hours. The absorbance was measured at 490 nm using a 96-well plate reader.

### 2.6. LDH Assay

In the lactate dehydrogenase (LDH) assay, cell injury was evaluated by measuring the leakage of LDH from impaired neurons. LDH activities in cell culture supernatants were examined as previously described [19]. The amounts of released LDH were determined by measuring the absorbance at a wavelength of 492 nm.

### 2.7. Western Blot Analysis

Equal amounts of protein extracts were loaded and separated by 5–12% SDS-PAGE gel and transferred onto PVDF membranes. After blocking with 5% skimmed milk, the membranes were incubated with the primary antibodies at 4°C overnight. Then alkaline phosphatase-conjugated secondary antibodies were adopted before treatment with the BCIP/NBT substrate solution. The western blot intensities were subsequently analyzed by Image J software.

### 2.8. Subcellular Fractionation

To determine the subcellular distribution of the AKAP79 in the cytoplasm and membrane fractions, cell lysates were prepared by ice-cold lysis buffer (10 mM Tris base, pH 7.6, 320 mM sucrose, 150 mM NaCl, 5 mM EDTA, 5 mM EGTA, 1 mM benzamidine, 1 mM 4-(2-aminoethyl) benzenesulfonyl fluoride, 2 g/mL leupeptin, 2 g/mL pepstatin, and 50 mM NaF) followed by homogenization. The homogenates were centrifuged at 960 ×g to remove nuclei and large debris (P1). The supernatants (S1) were fractionated into crude synaptosomal membranes (P2) and soluble supernatant/cytoplasm (S2) by 10,000 ×g for 30 min. The P2 pellet was homogenized in lysis buffer as above; aliquots of P2 and S2 were taken for western analysis [20].

### 2.9. Coimmunoprecipitation

Proteins were isolated from the cultured cells and 1000 *μ*g supernatants were transferred into each of microcentrifuge tubes. For preclearing the lysates, 1.0 *μ*g of control IgG and 20 *μ*L of Protein A/G-Agarose were added. Then 2 *μ*g of anti-AKAP79 primary antibody was placed in the tubes and incubated at 4°C overnight before the addition of 20 *μ*L of Protein A/G-Agarose. The mixture was incubated at 4°C on a rotating device overnight. The immunoprecipitates were collected by centrifugation and the pellets were gently washed with 1.0 mL RIPA buffer for twice to four times. The final pellets were resuspended with 40 *μ*L of 2x electrophoresis sample buffer and boiled for 5 minutes. Finally, the proteins were analyzed by western blot.

### 2.10. Immunofluorescence Assay

SH-SY 5Y cells were plated on chamber coverslips. After drug treatment, the chamber coverslips were gently washed with PBS, fixed in 3.7% paraformaldehyde for 15 min, permeabilized in PBS containing 0.1% Triton X-100 for 5 min, and blocked in PBS containing 5% BSA for 30 min. The fixed cells were incubated with anti-AKAP79 and anti-PKA RII antibodies overnight at 4°C and incubated with the DyLight 488 and DyLight 594 conjugated secondary antibodies for 2 h. After PBS wash, nucleus was stained by Hoechst 33342 for 10 min. The images were recorded by TCS-SP5 laser scanning confocal microscope (Leica Microsystems, Germany).

### 2.11. Statistical Analysis

The data shown were obtained from at least three independent experiments. The data in the different experimental groups are expressed as the mean ± S.E.M. Differences between the groups were evaluated by one-way ANOVA followed by Dunnett's test or Student-Newman-Keuls multiple comparison tests when necessary. Significant differences were represented as ^*∗*^ *P* < 0.05, ^*∗∗*^ *P* < 0.01, or ^*∗∗∗*^ *P* < 0.001.

## 3. Results

### 3.1. Pretreatment with Curcumin Attenuated Glutamate-Mediated Calcium Influx and Inhibited AKAP79 Translocation from Membrane to Cytoplasm

**Figure 1:**
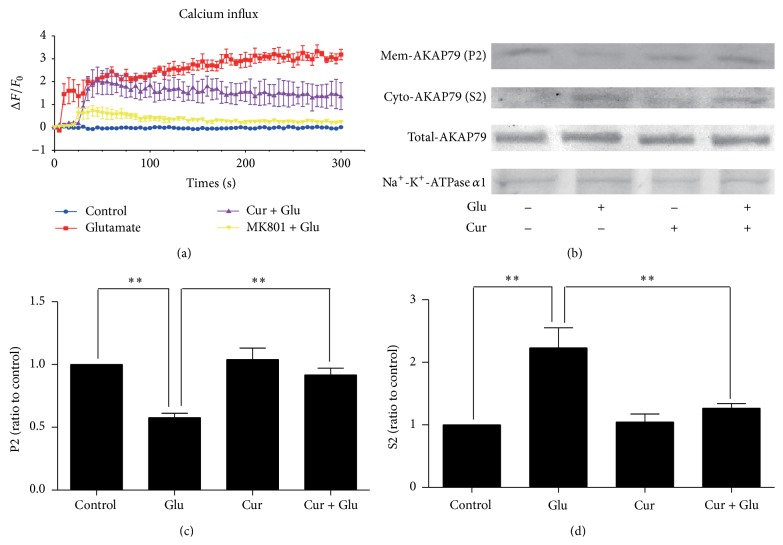
Curcumin blocked glutamate-mediated calcium influx and blocked the translocation of AKAP79-PKA complex. (a) Summary data describing the change of [Ca^2+^]_*i*_. (b) Glutamate induced an increase of AKAP79 in the S2 cytosolic fraction accompanied by decrease of AKAP79 in the P2 membrane fraction, which was blocked by curcumin. For membrane protein detection, commonly used internal reference protein is Na^+^-K^+^-ATPase. (c, d) Western blotting bands were quantified using Image J software. ^*∗∗*^ *P* < 0.01 versus glutamate group. (*n* = 3).

In this experiment, we examined [Ca^2+^]_*i*_ in control group, glutamate group, curcumin-pretreated group, and MK-801 group. The basal concentration of intracellular free Ca^2+^ was similar in all groups. In glutamate group, glutamate induced an obvious and long-term increase of calcium concentration. Unlike glutamate group, both the amplitude and the duration of Ca^2+^ influx were significantly attenuated in neurons of curcumin-pretreated group. Similarly, no calcium influx was observed in MK-801 pretreatment group (Figure 1(a)). In order to study the influence of calcium influx on the translocation of AKAP79-PKA complex, subcellular fractionation assay was conducted. As shown in Figures 1(b)–1(d), less AKAP79 was targeted to the plasma membrane after glutamate insult (*F*(3,8) = 14.39, *P* = 0.0014), while curcumin inhibited the redistribution of AKAP79-PKA complex from membrane to cytoplasm (*F*(3,8) = 10.52, *P* = 0.0038).

### 3.2. Curcumin Strengthened AKAP79-PKA Interaction Even after Glutamate Exposure

**Figure 2:**
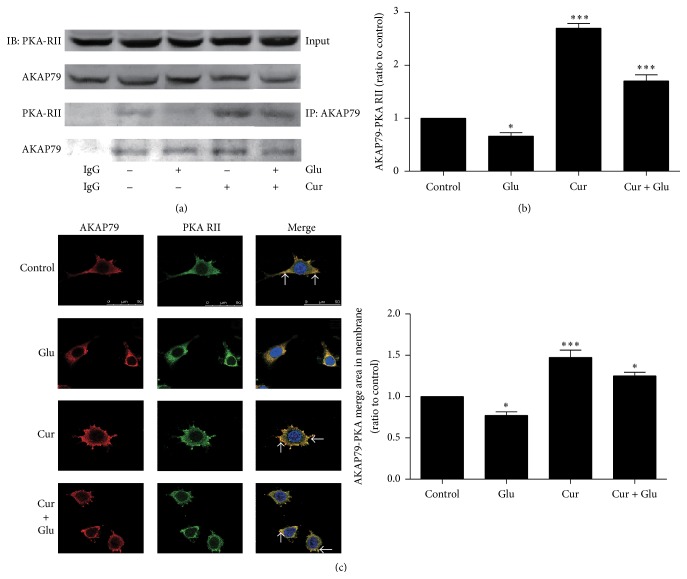
Curcumin enhanced the interaction between AKAP79 and PKA at the plasma membrane. (a) The change of AKAP79-PKA interaction after exposure to curcumin with or without glutamate exposure. (b) Western blotting bands were quantified using Image J software. (c) The colocalization changes between AKAP79 and PKA in membrane. The red fluorescent indicated the AKAP79 staining and the green fluorescent showed the PKA staining, while nucleus stained by Hoechst 33342 emitted blue fluorescent. The red fluorescent and green fluorescent merged into yellow staining which suggested the overlap between AKAP79 and PKA. Scale bar, 50 *μ*m. The colocalization of AKAP79 and PKA was quantified using Image J software. ^*∗*^ *P* < 0.05 and ^*∗∗∗*^ *P* < 0.001 versus control values. (*n* = 3).

Coimmunoprecipitation assay showed that glutamate apparently attenuated the interaction between AKAP79 and PKA (Dunnett's test, *q* = 2.989, *P* < 0.05), suggesting that glutamate was able to disturb the precise subcellular location of AKAP79-anchored PKA (Figures 2(a) and 2(b)). However, curcumin obviously strengthened AKAP79-PKA interaction (Dunnett's test, *q* = 15.08, *P* < 0.001) even after glutamate exposure (Dunnett's test, *q* = 6.259, *P* < 0.001) (Figures 2(a) and 2(b)). Immunofluorescence assay also found similar results for AKAP79-PKA interaction in membrane (Control versus Glu: Dunnett's test, *q* = 2.986, *P* < 0.05; Control versus Cur: Dunnett's test, *q* = 6.236, *P* < 0.001; Control versus Cur + Glu: Dunnett's test, *q* = 3.294, *P* < 0.05) (Figure 2(c)).

### 3.3. Curcumin Facilitated the Phosphorylation of AMPA Receptor but not NMDA Receptor

**Figure 3:**
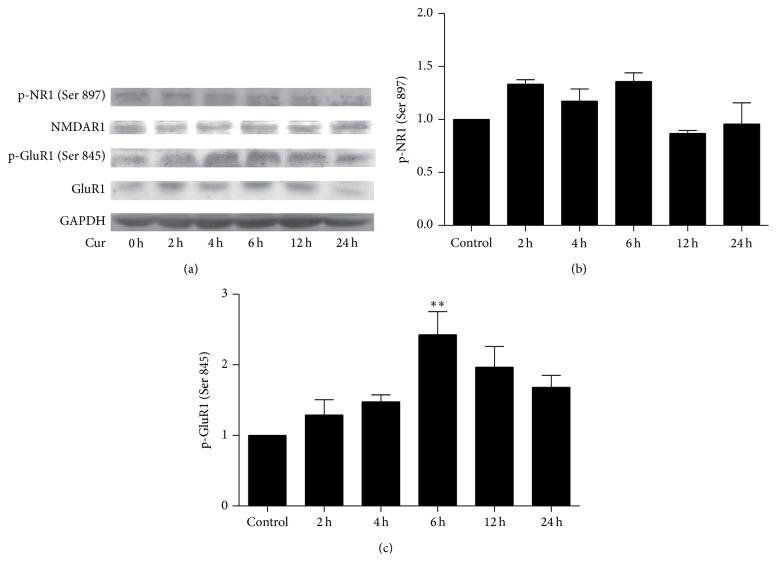
The time-course of curcumin on the phosphorylations of NR1 and GluR1. (a) Curcumin facilitated the phosphorylation of GluR1. GAPDH serves as the commonly used internal reference protein. (b, c) Western blotting bands were quantified using Image J software. ^*∗∗*^ *P* < 0.01 versus control values. (*n* = 3).

We further explored whether curcumin could influence the phosphorylation of AMPA receptor subunit GluR1 and NMDA receptor subunit NR1. No significant change was observed in p-NR1 (*F*(5,12) = 2.878, *P* = 0.062), while p-GluR1 level is enhanced for a long time especially after curcumin exposure for 6 hours (*F*(5,12) = 5.531, *P* = 0.0072) (Figure 3).

### 3.4. AKAP-Anchored PKA Was Crucial for Curcumin-Induced Activation of ERK and AKT Cascade

**Figure 4:**
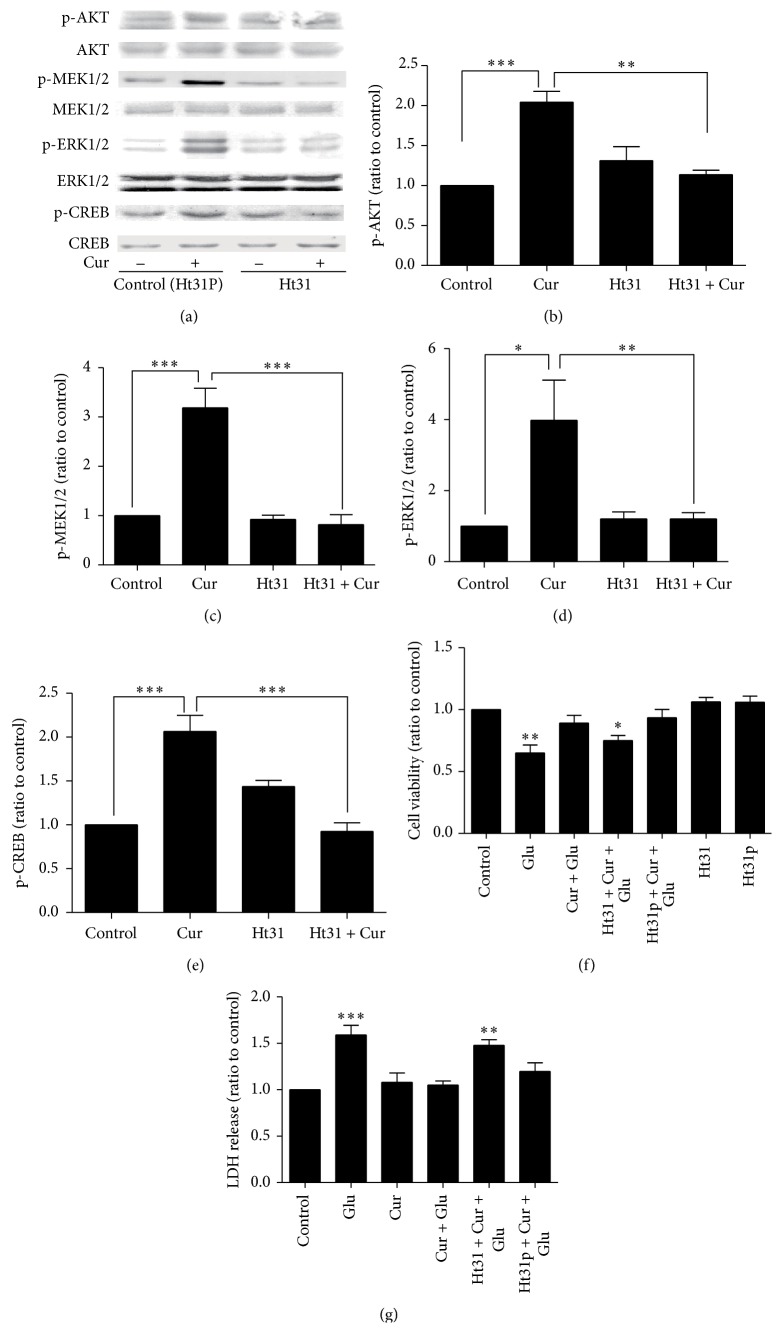
The neuroprotection of curcumin was abolished by PKA anchoring inhibitor Ht31. (a) Western blot analysis of the phosphorylations of AKT, MEK1/2, ERK1/2, and CREB. (b–e) Western blotting bands were quantified using Image J software. (f) Pretreatment with Ht31 suppressed curcumin-mediated increase of cell viability against glutamate insult. (g) LDH assay further confirmed the connection between PKA anchoring and curcumin-mediated neuroprotective effects. ^*∗*^ *P* < 0.05, ^*∗∗*^ *P* < 0.01, and ^*∗∗∗*^ *P* < 0.001 versus curcumin group (b–e) and versus control values (f, g). (*n* = 3).

Figures 4(a)–4(e) showed that phosphorylation of AKT (SNK test, *q* = 9.166, *P* < 0.001), MEK1/2 (SNK test, *q* = 9.766, *P* < 0.001), ERK1/2 (SNK test, *q* = 5.136, *P* < 0.05), and CREB (SNK test, *q* = 9.752, *P* < 0.001) was enhanced after curcumin treatment for 6 hours, while disruption of PKA anchoring with Ht31 attenuated curcumin-induced activations of ERK1/2 and AKT cascades.

As AKAP-anchored PKA was pivotal for neuronal survival related signaling pathway, we next performed experiments to determine whether curcumin-mediated neuroprotection was blocked by PKA anchoring inhibitor Ht31. MTS and LDH results showed that SH-SY 5Y neuronal cells presented a remarkable decrease in cell viability (Dunnett's test, *q* = 4.927, *P* < 0.01) and an increased LDH release (Dunnett's test, *q* = 5.457, *P* < 0.001) after exposure to 30 mM glutamate over 12 hours, while 1 *μ*M curcumin treatment for 6 hours displayed the effectively neuroprotective effect against glutamate excitotoxicity (MTS: Dunnett's test, *q* = 1.518, *P* > 0.05; LDH: Dunnett's test, *q* = 0.467, *P* > 0.05) (Figures 4(f) and 4(g)). Notably, no neuroprotective effect of the curcumin was observed if we administrated PKA anchoring inhibitor Ht31 in advance (MTS: Dunnett's test, *q* = 3.507, *P* < 0.05; LDH: Dunnett's test, *q* = 4.428, *P* < 0.01) (Figures 4(f) and 4(g)).

## 4. Discussion

In the present study, we have demonstrated that persistent and excessive glutamate insults triggered Ca^2+^ influx through NMDA receptor in SH-SY5Y human neuroblastoma cells, which is the key toxic mechanism of neurologic diseases, such as MDD, brain trauma, and Alzheimer's disease [21, 22]. To prove calcium response to glutamate was indeed mediated by NMDA receptor, we pretreated cells with NMDA receptor antagonist MK-801 and then exposed neurons to glutamate insult. Results showed that no calcium influx was observed in MK-801 pretreatment group. Similar to MK-801, curcumin also suppressed calcium overload in order to maintain cellular homeostasis for neuronal survival, which suggested that curcumin could antagonize NMDA receptor and block NMDA receptor-mediated calcium influx.

Recently, it has been presumed that PKA plays a critical role in antiexcitotoxic and antidepressant mechanisms [9, 10], and AKAP is the dominator to regulate the spatial and temporal organization of PKA as well as PKA related signal pathways [23]. As a main subunit of AKAP in SH-SY5Y neurons, scaffold protein AKAP79 is targeted to AMPA receptor in the postsynaptic density by interactions with cytoskeleton actin and PSD-95. Our study provides evidence that inhibition of Ca^2+^ influx by curcumin contributes to maintaining the membrane anchoring of AKAP79-PKA complex, and our results are in line with previous findings that blocking NMDA receptor-mediated calcium influx is in favor of maintaining the association of AKAP79 with membrane and AMPA receptor subunit GluR1 [20]. Increased [Ca^2+^]_*i*_ has been reported to induce remodeling of cytoskeleton and trigger the redistribution of signaling proteins bound to cytoskeleton from postsynaptic membranes to cytoplasm after glutamate insult [20]. As Smith reported [20], glutamate disrupted the precise anchoring of AKAP79-PKA compound leading to AKAP79 loss from postsynaptic membrane in our study. Unexpectedly, glutamate also reduced AKAP79-PKA interaction to target even less PKA around AMPA receptor. On the contrary, curcumin retained more AKAP79-PKA complex on the neuronal membrane and augmented AKAP79-PKA interaction.

What is the significance of targeting more AKAP79 compounds around GluR1 and enhancing AKAP79-PKA interaction after curcumin treatment? AKAP79-anchored PKA is reported to phosphorylate GluR1 to increase the open probability of AMPA receptor and further relay signals to downstream pathways via PI3K-dependent activation of MAPK [24, 25]. After AKAP79/150 knockout, Ser-845 phosphorylation of GluR1 is significantly reduced which highlights the strong impact of AKAP79/150 and AKAP-anchored PKA on the function of GluR1 [26]. Thus, we hypothesized that curcumin could influence the phosphorylation of GluR1 or NR1. As we expected, increased phosphorylation of the GluR1 was found after 1 *μ*M curcumin pretreatment, while there was no remarkable difference for p-NR1, which suggested that enhanced phosphorylated GluR1 may participate in curcumin-mediated neuroprotective process.

AMPA receptor-mediated upregulation of AKT and ERK cascades is reported to be crucial for NMDA receptor antagonist-induced rapid antidepressant effects [27], so we further investigated whether ERK and AKT cascades were involved in curcumin-mediated neuroprotective effects. Enhanced AKT and ERK cascades were observed after curcumin treatment. Importantly, the signal transmitted by AKT and ERK cascades further resulted in upregulation of p-CREB. CREB is a cellular transcription factor which binds to cAMP response elements (CRE) sequence of DNA to manipulate the transcription of the downstream genes, such as BDNF gene. CREB phosphorylation at serine 133 is important for the survival of neurons [28]. Interestingly, activation of AKT and ERK cascades is in PKA-dependent manner. If we pretreated neurons with PKA anchoring inhibitor Ht31, no activation of ERK1/2 and AKT cascades was observed. Accordingly, MTS and LDH assays confirmed that curcumin did rescue neurons from glutamate insult in PKA-dependent manner which indicated that AKAP79-anchored PKA is crucial for curcumin to resist glutamate insult in MDD cell models. Our results are consistent with previous findings that AKAP-anchored PKA can facilitate signal relay from MEK1/2 to ERK1/2 [29]. Additionally, our observations are also in line with the evidence that PKA is required for AKT phosphorylation [30]. It should be noted that Ht31 is a nonselective PKA anchoring inhibitor; other AKAP subunits may also be involved in curcumin-mediated neuroprotective effects.

In conclusion, the present study provides direct evidence that AKAP79-PKA complex participates in the antidepressant mechanisms of curcumin. Targeting more AKAP79-anchored PKA to membrane is in favor of activating GluR1 and its downstream pathways, such as AKT and ERK cascades. Thus AKAP79-PKA complex can serve as potential drug targets to regulate anchored signaling protein and their downstream pathways, which have implications for research and development of novel antiexcitotoxic and antidepressant drugs.

## Abbreviations

MDD: Major depressive disorder
NMDA receptor: N-Methyl-D-aspartic acid receptor
AMPA receptor: *α*-Amino-3-hydroxy-5-methyl-4-isoxazolepropionic acid receptor
AKAP: A-kinase anchoring proteins
PKA: Protein kinase A
CUMS: Chronic unpredictable mild stress
CREB: cAMP response element-binding protein
CRE: cAMP response elements
LDH: Lactate dehydrogenase
MTS: 3-(4,5-Dimethylthiazol-2-yl)-5-(3-carboxymethoxyphenyl)-2-(4-sulfophenyl)-2H-tetrazolium
MEK: Mitogen-activated protein kinase
ERK: Extracellular regulated kinase
ANOVA: Analysis of variance.
